# Mortality and associated risk factors for older adults admitted to the emergency department: a hospital cohort

**DOI:** 10.1186/s12877-018-0833-0

**Published:** 2018-06-18

**Authors:** Carmen García-Peña, Mario Ulises Pérez-Zepeda, Leslie Viridiana Robles-Jiménez, Sergio Sánchez-García, Ricardo Ramírez-Aldana, Pamela Tella-Vega

**Affiliations:** 1 Dirección de Investigación, Instituto Nacional de Geriatría, Periférico Sur No. 2767, Mexico City, Mexico; 2 Instituto Nacional de Psiquiatría Ramón de la Fuente Muñiz, Mexico City, Mexico; 3 Unidad de Investigación en Epidemiología y Servicios de Salud, Área Envejecimiento, Mexico City, Mexico; 4 Facultad de Ciencias. Universidad Nacional Autónoma de México, Mexico City, Mexico

**Keywords:** Acute care, Emergency medicine, Geriatrics, Mortality

## Abstract

**Background:**

Older emergency department patients are more vulnerable than younger patients, yet many risk factors that contribute to the mortality of older patients remain unclear and under investigation. This study endeavored to determine mortality and factors associated with mortality in patients over 60 years of age who were admitted to the emergency departments of two general hospitals in Mexico City.

**Methods:**

This is a hospital cohort study involving adults over 60 years of age admitted to the emergency department and who are beneficiaries of the Mexican Institute of Social Security and residents of Mexico City.

All causes of mortality from the time of emergency department admission until a follow-up home visit after discharge were measured. Included risk factors were: socio-demographic, health-care related, mental and physical variables, and in-hospital care-related. Survival functions were estimated using Kaplan-Meier curves. Hazard ratios (HR) were derived from Cox regression models in a multivariate analysis.

**Results:**

From the 1406 older adults who participated in this study, 306 (21.8%) did not survive. Independent mortality risk factors found in the last Cox model were age (HR = 1.02, 95% CI, 1.005–1.04; *p* = 0.01), length of stay in the ED (HR = 1.003, 95% CI = 0.99, 1.04; *p* = 0.006), geriatric care trained residents model in Hospital A (protective factor) (HR = 0.66, 95% CI = 0.46, 0.96; *p* = 0.031), and the FRAIL scale (HR of 1.34 95% CI, 1.02–1.76; *p* = 0.033).

**Conclusions:**

Risk factors for mortality in patients treated at Mexican emergency departments are length of stay and variables related to frailty status.

## Background

Around the world, aging populations pose major challenges to societies and their health care systems. The health needs of older individuals will continue to grow and have greater impact on the financial, social, and health systems of most countries, with developing countries experiencing the greatest impact. This will create an unprecedented situation for our global society [1]. The health care requirements of aging individuals are broad, ranging from ambulatory clinical services, home health visits, and acute hospitalization to long term and palliative care [2]. Health care systems are not responding effectively or efficiently [3] because they are not equipped to handle the increased needs of older adults, particularly since they were designed to treat the injuries or acute medical conditions of a younger population [4].

Existing literature reveals that older adults may experience increased vulnerability [5] in emergency departments (ED) because of a deficient response in services [6]. Global Aging and Adult Health (SAGE) [7] collected data via nationally representative population surveys in six middle-income countries: China, Ghana, India, Mexico, Russia, and South Africa. The data reported a prevalence of multimorbidity of 29.6% in the 70+ age group in Mexico, one of the highest rates among the populations studied. Additionally, in the six countries included in the study, individuals who had been hospitalized over the past three years had a higher likelihood of contracting non-communicable diseases. A separate study also reported that adults over the age of 60 accounted for between 13 and 18% of visits to the ED in Mexico [8]. Data from other studies shows that 14% of ED visits correspond to the 60+ age group [9, 10].

A combination of intrinsic and extrinsic factors work together to increase the mortality rate of this age group [11]. One of the main factors is the ability of health care workers to effectively assess the risk factors associated with mortality; a complex picture driven by the heterogeneous patterns of morbidity of this age group and the interactions with conditions particular to aging that preclude or delay risk stratification or that make assessment less accurate than in other age groups [12]. Older adults seek care in the ED for varied reasons and very often they present interacting conditions, a result of the convergence of multimorbidity with so-called geriatric syndromes (delirium, cognitive impairment, falls, incontinence, abuse, and mobility disability) [13].

Nevertheless, it is undeniable that identifying the risk factors for mortality in older adults could guide and individualize interventions for those patients most in need [14]. In fact, exposure to the ED and subsequent hospitalization may itself be considered a risk factor. Adults older than 65 years represent nearly half of the mortality cases in EDs [15, 16]. As stated by de Decker et al., an emergency department is not only a place to care for old people, but also a place to die [17]. Therefore, it is imperative to determine which specific risk factors may contribute to the mortality of older adults who visit the ED.

In this study, we aimed to determine mortality and the associated factors in older adults (> 60 years of age) who were admitted to the ED in two general hospitals in Mexico City. Both hospitals were part of the *Instituto Mexicano del Seguro Social (Mexican Institute of Social Security, or* IMSS) system.

## Methods

This report was based on a quasi-experimental study named “Old Persons in the Emergency Services of General Hospitals: Effectiveness of a Geriatric Care Training Model for Residents to Improve Health Results,” registered at clinicaltrails.gov (NCT01706133). Two general hospitals were included and a training model was carried out in one of them. Basal and final cohorts of patients were integrated. The main aim of the study was to test an intervention based on geriatric care training of emergency care resident physicians in order to reduce frailty levels. In this present report, we considered all patients included. The geriatric care training model was only analyzed as an additional explanatory variable for mortality.

The eligible population was made up of adults over 60 years of age who visited the ED in one of two IMSS general hospitals in Mexico City. IMSS is a mandatory social security system that offers a comprehensive package of benefits to roughly half of the population in Mexico, including healthcare at all levels, as well as social and economic benefits (e.g., retirement pensions). IMSS covers the needs of non-governmental workers and their families. The IMSS health care system assigns each individual and immediate family member to a Family Medicine Unit, which is the primary health care provider, with secondary and tertiary health care provided as needed based on referrals from the Family Medicine Unit.

Participants were included in the study after being evaluated by a physician in triage and subsequently being admitted to the ED. The research team did not interfere with the medical decisions made in triage nor in the ED admission process. Patients with an imminent, acute, life-threatening condition that required immediate attention (intensive care), victims of a car accident, or patients who suffered second- or third-degree burns were not included.

The sample selection process was done consecutively, with the sample size calculated for the intervention project with an expected difference of 20% between the proportion of basal and final frailty. A lowering in frailty levels was the outcome of interest, with an alpha of 0.05 and a beta of 0.20 for a total of 480 patients and an additional 20% for possible loss during follow-up. The total sample size was included in the present study. This sample size was sufficient to determine mortality risk factors, which is the aim of the present report.

The main outcome was to determine overall mortality (all causes of death), independent of the site of occurrence (e.g., in the emergency department, post-ED hospitalization, or at home after discharge). Research staff collected this information at the hospital during the ED visit or post-ED hospitalization, or with next-of-kin at home. The first day of survival was recorded as the date admitted to the ED and the final follow-up was at 120 days after the visit to the ED. We chose 120 days as the cut off for follow up because we believe 120 days gave us enough timing to gather valid statistics while taking into consideration that the cut off for follow up also needed to be close enough to the date of ED admission for any deaths to be attributable to the ED visit.

In order to assess mortality risk factors, a set of independent variables from different dimensions was explored: the individual dimension included socio-demographic aspects such as age, sex, education, marital status, living condition, type of insurance, and financial position. A second dimension of variables was related to the health service provided during the current event and included: waiting time (in hours) in the ED, the number of previous visits to the ED (related or not to the medical reason for the current ED visit) during the six months prior to the current visit, the number of visits to the Family Medicine Clinic (prior to the current ED visit and for the same medical reason), whether there was a delay in seeking care at the ED for any reason (yes or no), the length of stay (in hours) in the ED, and finally, the model of residents trained in geriatric care (intervention) or referral for hospitalization in the same facility. The dimension of health-related variables included: main diagnosis upon admission, whether there was a presence of delirium (evaluated through the Confusion Assessment Method [CAM] [18], the current level of pain (evaluated with a 10-point Visual Analogue Scale for Experimental Pain with 10 being a maximum) [19], handgrip strength (assessed with a handheld electronic dynamometer adjusted for gender, with a cutoff mark of 17 kg for women and 30 kg for men) [20], whether the individual had suffered falls within the last year (yes or no), the total number of medications currently taken, the presence of depressive symptomatology (measured with the Brief Depression Screen developed for older people in ED) [21] and finally, morbidity (summatory of chronic diseases). Additional health-related variables included whether there was cognitive impairment, assessed with a valid Spanish-language version [22] of the Folstein [23] Mini-Mental State Examination (MMSE). The MMSE evaluates memory, orientation to space and time, ability to perform calculations, language and word recognition, with scores ranging from 0 to 30 points and lower scores indicating poorer cognitive ability. The cut off point for inclusion in the study was ≤23, adjusted by age. Functionality was measured with the Barthel index [24] where a score of 100 means independence. The risk of adverse future events index was measured by the Identification of Seniors at Risk (ISAR) scale [25], with a positive score of 2 or more from a total of 6 points. Finally, the frailty status of participants was measured with the FRAIL scale, composed of 5 items (fatigue, resistance, ambulation, illness, and loss of weight) frailty considered present with 2 or more points [26].

### Procedures

Once admitted to the ED by hospital staff, each patient was evaluated by properly trained nurses from the research team to determine whether the patient met the selection criteria. If so, written consent was obtained, baseline measurements were taken, and a questionnaire was completed within the first two hours of admission to the ED. Patients were monitored throughout their ED stay by the research nursing team to record if the hospital staff decided to discharge or hospitalize the patient and to record the participants’ vital signs. A follow-up evaluation was conducted in participants’ homes 120 days after admission to the ED. In cases where participants had died, primary caregivers, as proxies, were interviewed for the follow-up. The questionnaires were processed and reported by third parties who were not involved in the project and therefore did not possess any knowledge of the study. The data collection was carried out from June, 2013 to February, 2014.

### Ethical issues

The research protocol was approved by the Ethics and Scientific Commission of IMSS (R2011–785-056). An informed written consent was signed by each willing participant or by the participant’s representative. It was made clear that patients were free to refuse to take part in the study or to withdraw from the study at any point by submitting a simple request. Patients were also advised that they would experience no consequence or alteration to the medical care provided if they refused to participate or withdrew after having previously begun to participate.

### Statistical analysis plan

Censored cases included surviving individuals and those who dropped out of the study or were excluded, and uncensored included those patients who died. Survival time was determined from the date the patient was admitted to the ED for initial observation [27] up to 120 days later. The final time for uncensored individuals represented the date of death at any time between admission for observation and the study end date, which corresponded to the period in which evaluation was performed at home. For censored individuals, the end time was the date of the final home visit because, although, there were individuals who withdrew from the study and therefore did not fill out the questionnaires, they were determined to be alive. There were cases in which information was obtained after the end of the study, however 120 days was considered as the cutoff time for all survival analysis. Considering this information and five additional cases (4 were patients who died) without recorded dates, 48 cases were removed from the survival analyses. Cases were removed based on missing values in the involved variables and no data input was performed on those cases.

Descriptive and bivariate analyses (between the censored and uncensored patients) was performed according to the type of variable. If the variable was nominal or ordinal, a chi-squared test was performed; if the variables were qualitative or ordinal with multiple values, t-tests were used; if normality was inadequate, the Mann-Whitney test and medians were used.

Survival functions were estimated through Kaplan-Meier curves (KMC). To determine significant differences, comparisons of survival curves were performed applying log-rank tests. To conduct the comparisons, qualitative variables were used when possible, and, when necessary, new categorized variables were obtained from quantitative variables [27].

Age groups, years of formal education groups (0, 1–6, 7–12 and > 12) and handgrip strength (categorized as abnormal according to the description in the methods section) in the ED on completion of the KMC were included.

Those variables that were significantly different according to vital status were selected to perform a Cox regression (27). The models were simplified according to the significance of the variables, leaving only some control variables (e.g., age and sex). The effect of the intervention-trained residents for the geriatric care model (see above intervention) was added as a factor, which was obtained by taking into consideration the hospital in which the intervention was carried out.

## Results

**Fig. 1:**
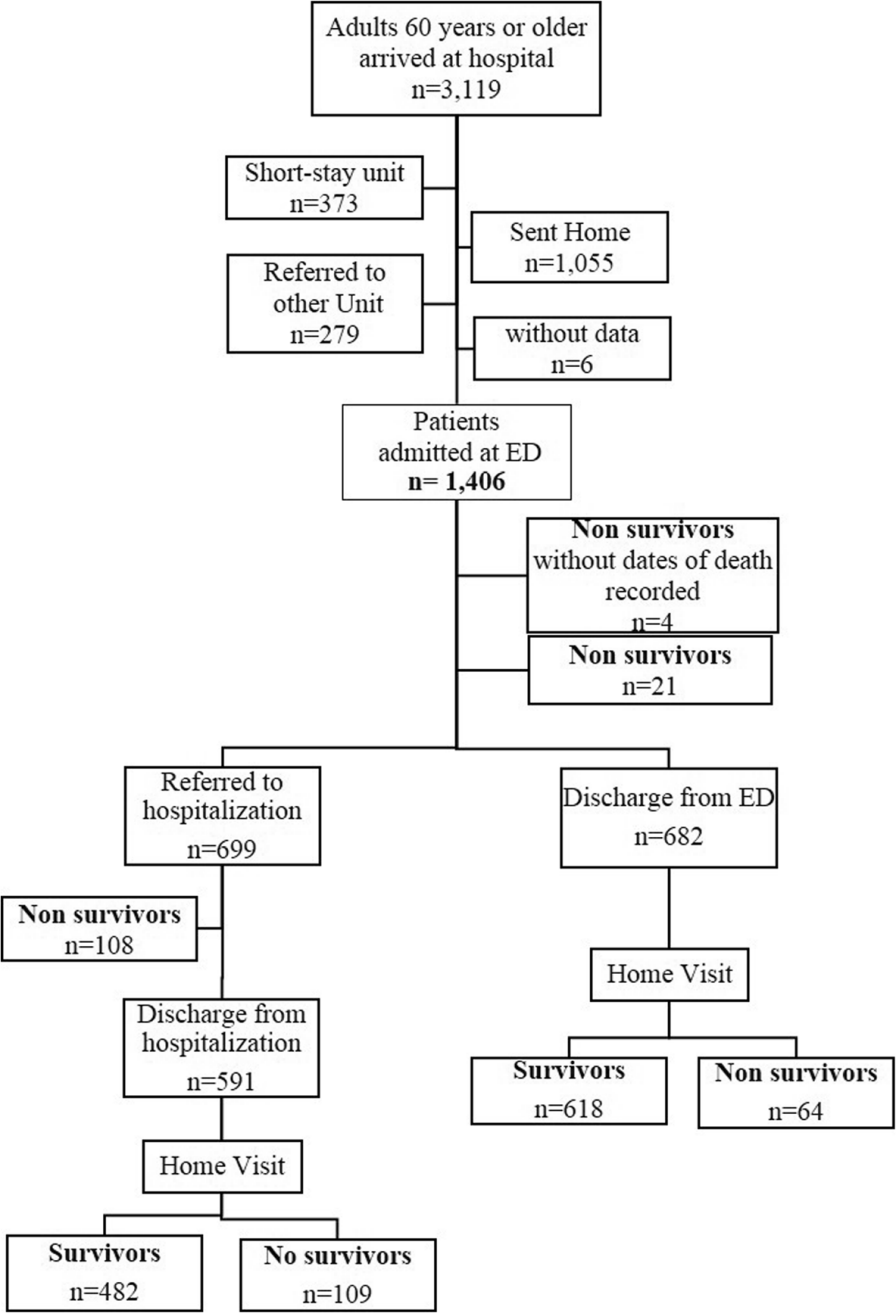
Flow chart of the study participants

Flow of study participants is presented in Fig. 1. During the study period (from June, 2013 to February, 2014), a total of 3119 patients 60 years and older arrived in the ED. Of these, 1055 were checked by the social worker at reception triage and sent back to their home, 279 were referred to another hospital, and 373 were admitted to an area named “short stay unit” (a consultation room for minor procedures such as bandaging, receiving a prescribed medication injection, minor wound healing, or any other non-life threatening condition). No participant was eliminated due to an acute, life-threatening condition because such conditions are not treated in general hospitals such as the hospitals included in the study. This study evaluated a total of 1406 adults 60-years or older who were admitted to EDs in one of two hospitals. *N* = 705 in Hospital A (50.14%) and *n* = 701 in Hospital B (49.86%). From the final sample, 59.17% (*n* = 832) were women. The average number of years of school attendance was 6.31 (± standard deviation [SD] of 4.9) years; about 43.92% of participants were married or cohabiting; and 10.39% (*n* = 142) lived alone. Regarding financial status, about 46.52% (*n* = 588) of patients reported that their income barely covered their financial needs. As for the factors associated with the health care process, the average waiting time between arrival and admission was 4.8 h (± SD 5.94), 41.4% of older adults had not previously gone to an ED, and in the case of recurrence of the same symptoms or medical reason, 37.67%) (*n* = 524) delayed seeking attention at the ED. The average stay in the ED was 100.79 (± 67.2) hours and 699 subjects (49.7%) were hospitalized to a general ward after ED admission (Table 1).

**Table 1:** Bivariate analysis of survival by variable categories

Variables	Nonsurvivors *n* = 306 (21.8%)		Survivors *n* = 1100 (78.2%)		TOTAL *n* = 1406		*P* value
	Sociodemographic						
Age, mean (SD)	78.2	(8.3)	76.3	(7.8)	76.7	(7.9)	0.002
Sex, n (%)	131	(22.8)	443	(77.2)	574	(40.8)	0.424
Man							
Woman	175	(21.0)	657	(79.0)	832	(59.2)	
Schooling, mean (SD)	6.9	(4.9)	6.2	(4.9)	6.3	(4.9)	0.039
Civil status, n (%)							
Married	124	(21.5)	454	(78.5)	578	(43.9)	0.229
Widower	108	(19.7)	439	(80.2)	547	(41.6)	
Other	49	(26.7)	142	(74.3)	191	(14.5)	
Lives alone, n (%)	32	(22.5)	110	(77.5)	142	(10.4)	0.718
Financial situation, n (%)							
I do not have money problems	103	(20.6)	395	(79.4)	498	(39.4)	0.29
It is difficult to cover my expenses	132	(22.5)	456	(77.5)	588	(46.5)	
My expenses are too much, I cannot cover them	32	(17.9)	146	(82.0)	178	(14.1)	
	Health Service						
Waiting time in the ED, mean, hours (DE)	4.45	(5.9)	4.9	(5.9)	4.8	(5.9)	0.298
Number of visits to ED, n (%)							
None	124	(22.4)	430	(77.6)	554	(41.4)	0.775
Once	84	(21.7)	303	(78.3)	387	(28.9)	
Twice or more	81	(20.4)	315	(79.5)	396	(29.6)	
Visits to the Family Medicine Clinic, median (IQR)	5	(0–20)	5	(0–25)	5	(0–25)	0.42
Delayed in seeking attention at ED, n (%)	99	(18.9)	425	(81.1)	524	(37.7)	0.048
Length of stay (in hours) in ED, mean, hours (SD)	111.6	(63.2)	97.84	(68.0)	100.8	(67.2)	< 0.001
Hospital, n (%)							
A	151	(21.4)	554	(78.6)	705	(50.1)	0.753
B	155	(22.1)	546	(77.9)	701	(49.9)	
Geriatric care trained residents, n (%)							
Yes	67	(19.4)	279	(80.6)	346	(24.6)	
No	239	(22.5)	821	(77.5)	1060	(75.4)	0.213
Referral to hospitalization, n (%)							
Yes	217	(31.0)	482	(69.0)	699	(49.7)	0.000
No	89	(12.6)	618	(87.4)	707	(50.3)	
	Health						
Admission reason, n (%)							
Anorexia/weight lost	2	(22.2)	7	(77.8)	9	(1.01)	0.053
Neurological problems	15	(18.1)	68	(81.9)	83	(5.9)	
Associated diabetes problems	3	(13.0)	20	(86.9)	23	(1.6)	
Cardiovascular problems	12	(14.6)	70	(85.3)	82	(5.8)	
Pneumological problems	28	(28.8)	69	(71.13)	97	(6.9)	
Gastrointestinal problems	55	(22.0)	195	(78.0)	250	(17.8)	
Genitourinary problems	8	(17.4)	38	(82.6)	46	(3.3)	
Nonspecific symptoms	28	(15.9)	148	(84.1)	176	(12.5)	
Procedures/other services	7	(13.4)	45	(86.6)	52	(3.7)	
Other reasons	15	(20.8)	57	(79.2)	72	(5.1)	
Nonspecified	133	(25.7)	383	(74.3)	516	(36.7)	
Number of admission reasons, median (RIC)	2	(1–7)	2	(1–6)	2	(1–7)	0.69
Delirium scale changes, n (%)							
Improved	80	(26.5)	222	(73.5)	302	(21.5)	< 0.001
Same	202	(19.3)	845	(80.7)	1047	(74.5)	
Worsened	24	(42.1)	33	(57.9)	57	(4.0)	
Visual analogue scale of pain, mean (SD)	3.39	(3.7)	3.57	(3.9)	3.54	(3.7)	0.475
Grip strength, mean (SD)	3.37	(5.6)	4.81	(7.1)	4.49	(6.8)	0.001
Falls, n (%)	180	(21.5)	657	(78.5)	837	(59.5)	0.776
Number of medications, median (IQR)	3	(0–12)	3	(0–16)	3	(0–16)	0.112
Feeling sad in the last two weeks, n (%)	128	(20.8)	485	(79.2)	613	(68.5)	0.221
Charlson index, median (RIC)	4	(1–13)	6	(5–10)	4	(1–13)	0.896
MMSE, mean (SD)	17.8	(7.0)	19.4	(6)	19.41	(6.3)	< 0.001
Barthel index, mean (SD)	63.32	(34.2)	72.16	(28.6)	70.29	(30.1)	< 0.001
ISAR, n (%)	198	(17.1)	960	(82.9)	1158	(82.4)	0.001
FRAIL, n (%)	134	(21.8)	480	(78.2)	614	(45.2)	< 0.001

The main reason for emergency admission was gastrointestinal problems (17.78%), followed by nonspecific symptoms (12.52%). An average score of 3.54 (± SD 3.69) was reported in the visual analog scale of pain (0–10). Mean handgrip strength was 4.49 kg (± SD 6.81). The report of at least one fall within the previous year occurred in 59.53% of patients (*n* = 837). The median number of medications was three (interquartile range [IQR] = 0–16), whereas the median number of the sum of chronic diseases was four. Among study participants, 68.49% reported depressive symptoms. The Barthel index had an average score of 70.29 (±SD 30.08), and the average score of the Mini-mental State Examination (MMSE) was 19.41 (± SD 6.32). A total of 82.36% of study participants had two or more points (high risk) using the ISAR tool and 45.18% were scored as frail using the FRAIL scale (see Table 1). Finally, 49.7% patients (*n* = 699) were hospitalized (internal medicine wards).

The bivariate analysis for mortality for the sociodemographic variables showed significant differences for age [78.2 years (±SD 8.3) and 76.3 years (±SD 7.8)] for non-survivors and survivors, respectively (*p* = 0.002). Factors associated with the healthcare process were statistically significant for delayed arrival to the ED (*p* = 0.048), the number of hours in the ED [111.62(±SD 63.18) for non-survivors versus 97.84(±SD 68) for survivors](*p* < 0.001), and any referral to hospitalization (*p* = < 0.001). Hand grip strength was significantly higher in survivors (4.81 vs 3.37, *p* = 0.001). Regarding the MMSE, the mean score was (19.41 vs 17.8, *p* < 0.001) and the Barthel index mean score was (72.16 vs 63.32, *p* < 0.001). There was also a significant difference between survivors and non-survivors. Finally, both the ISAR and FRAIL scale proportions were significantly different between survivors and non-survivors during the follow-up period (Table 1).

**Fig. 2:**
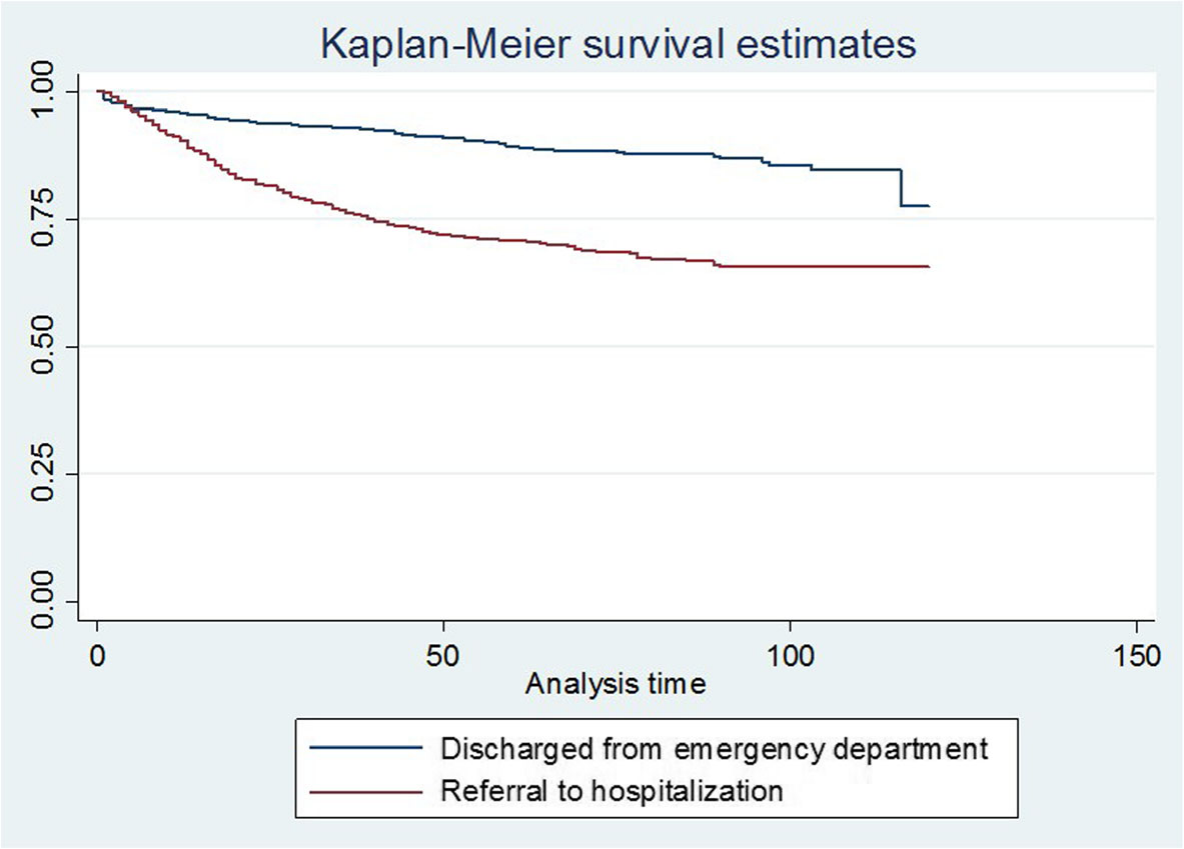
Kaplan-Meier Curves and *Log-rank* tests for group referred to hospitalization versus discharged from ED

Regarding the survival analysis, variables which were statistically significant according to the bivariate analysis are shown in the different KMC curves (Fig. 2). Those variables with a *p*-value < 0.2 in the bivariate analysis were included in a Cox regression unadjusted model and three models were analyzed: all patients, patients not referred to hospitalization (*n* = 682), and patients referred to hospitalization (*n* = 699) in which age, length of stay in the ED, and referral to hospitalization were significant. Neurological problems were also significant except in the group with all patients (Table 2).

**Table 2:** Cox regression (variables included: those with significance < 0.2 in the bivariate analysis, as well as sex and geriatric care trained residents)

Variable	All patients			Discharged from emergency department *n* = 682			Referral to hospitalization *n* = 699		
	HR (95% CI)		Significance	HR (95% CI)		Significance	HR (95% CI)		Significance
Age	1.03	(1.00-1.05)	0.007	1.04	(1.00–1.09)	0.04	1.02	(0.99–1.05)	0.38
Woman	0.75	(0.52–1.07)	0.117	0.45	(0.22–0.90)	0.02	0.82	(0.52–1.28)	0.38
Schooling	1.03	(0.99–1.06)	0.145	1.00	(0.93–1.08)	0.79	1.03	(0.98–1.07)	0.14
Length of stay in ED	1.00	(1.00–1.01)	< 0.001	1.00	(0.99–1.00)	0.12	1.01	(1.00–1.01)	0.001
Delayed in seekig attention at ED	0.79	(0.54–1.15)	0.22	1.99	(0.99–3.98)	0.05	0.52	(0.31–0.86)	0.001
Referral to hospitalization	2.79	(1.89–4.10)	< 0.001	–	–	–	–	–	–
Geriatric care trained residents model (Hospital A final)	0.64	(0.39–1.11)	0.115	1.02	(0.42–2.47)	0.95	0.64	(0.33–1.24)	0.18
Health problems:									
Anorexia/weight loss	4.86	(0.49–48.07)	0.176	2.92e-15	(0–0)	1.00	60.64	(4.61–796.77)	0.002
Neurologic	0.80	(0.32–2.04)	0.653	5.88	(1.73–19.89)	0.004	0.18	(0.02–1.36)	0.09
Diabetes-associated	1.39	(0.43–4.50)	0.587	0.69	(0.08–5.68)	0.73	1.61	(0.37–7.00)	0.52
Cardiovascular	1.11	(0.52–2.34)	0.791	1.45	(0.18–11.37)	0.72	1.15	(0.50–2.61)	0.73
Pneumological	1.68	(1.03–2.75)	0.037	1.07	(0.34–3.31)	0.90	1.66	(0.94–2.93)	0.07
Gastrointestinal	1.04	(0.64–1.68)	0.879	1.09	(0.46–2.60)	0.83	0.87	(0.47–1.60)	0.67
Genitourinary	0.20	(0.03–1.42)	0.107	2.16e-15	(0–0)	0.90	0.29	(0.03–2.15)	0.22
Delirium scale changes, n (%)									
Same	Reference								
Improved	0.81	(0.39–1.68)	0.581	0.69	(0.19–2.47)	0.57	0.63	(0.25–1.60)	0.34
Worsened	1.61	(0.55–4.73)	0.383	1.16	(0.13–10.03)	0.89	2.21	(0.63–7.76)	0.21
Grip strength	0.97	(0.94–1.00)	0.101	1.00	(0.96–1.05)	0.71	0.93	(0.88–0.98)	0.007
Number of medications	1.05	(0.99–1.12)	0.108	1.17	(1.03–1.33)	0.01	1.03	(0.95–1.11)	0.37
Mini-mental state examination score	0.98	(0.95–1.01)	0.291	0.97	(0.90–1.04)	0.41	0.99	(0.95–1.03)	0.38
Barthel index score	0.99	(0.99–1.00)	0.171	0.99	(0.98–1.01)	0.67	0.99	(0.98–1.00)	0.14
FRAIL dichotomic (≥2)	0.93	(0.63–1.39)	0.738	0.43	(0.18–1.03)	0.06	1.18	(0.73–1.89)	0.48
ISAR dichotomic (≥2)	1.11	(0.63–1.96)	0.707	2.05	(0.57–7.32)	0.26	0.83	(0.43–1.60)	0.59

A simplified Cox model was obtained using all significant variables in the “all patients” model given in Table 2, including the intervention and other variables significant at a 0.1 level (Table 3). Considering the hazard ratio (HR) of death in the model with all patients, we observed that age remained significant (HR = 1.02 confidence interval [CI] 95% 1.004–1.04, *p* = 0.014), and education level was significant (HR = 1.04, CI 95% 1.00–1.07, *p* = 0.020). HR for length of stay was 1.004 (CI 1.002–1.006, *p* < 0.01), grip strength was 0.97 (CI 95% 0.95–1.00, *p* = 0.052), and the MMSE score was 0.97 (CI 95% 0.95–0.99, *p* < =0.001). HR for residents trained for geriatric care intervention was 0.70, with a marginal significance level in the limit, (CI 95% 0.48–1.02, *p* = 0.06) (Table 3). In the model of those subjects without referral to be hospitalized, only the MMSE maintained significance (HR = 0.94 CI 95% 0.90–0-99, p = 0.02). In contrast, for those with referral to be hospitalized, significant variables were age, education level, length of stay in the ED and handgrip strength (Table 3).

**Table 3:** Simplified Cox regression model

Variable	With only significant variables			Discharged from emergency department *n* = 682			Referral to hospitalization *n* = 699		
	HR (95% CI)		Significance	HR (95% CI)		Significance	HR (95% CI)		Significance
Age	1.02	(1.00–1.04)	0.014	1.03	(0.99–1.06)	0.073	1.02	(0.99–1.04)	0.05
Schooling	1.04	(1.01–1.07)	0.020	1.01	(0.95–1.07)	0.61	1.04	(1.00–1.08)	0.02
Length of stay in ED	1.00	(1.00–1.01)	0.001	1.00	(0.99–1.00)	0.469	1.00	(1.00–1.01)	0.001
Referral to hospitalization	2.86	(2.08–3.92)	< 0.000	–	–	–	–	–	–
Geriatric care trained residents model (Hospital A final)	0.70	(0.48–1.03)	0.067	1.02	(0.41–1.71)	0.636	0.66	(0.42–1.04)	0.076
Grip strength	0.97	(0.95–1.00)	0.052	1.01	(0.97–1.05)	0.45	0.95	(0.92–0.99)	0.009
Mini-mental state examination score	0.97	(0.95–0.99)	0.000	0.94	(0.90–0.99)	0.02	0.98	(0.95–1.05)	0.115

In a third simplified Cox model considering all patients, age was also significant, as was length of stay in the ED, the presence of residents trained for geriatric care intervention, and the FRAIL scale, which obtained an HR of 1.34 (95% CI, 1.02–1.76; *p* = 0.033) (Table 4). Age was the only significant variable in the model including only patients not referred for hospitalization (HR = 1.04 CI95% 1.002–1.069, p = 0.03) and all variables were significant in the model with patients referred to hospitalization (Table 4).

**Table 4:** Regression Model for mortality^a^

Variable	With only significant variables			Discharged from emergency department *n* = 682			Referral to hospitalization *n* = 699		
	HR (95% CI)		Significance	HR (95% CI)		Significance	HR (95% CI)		Significance
Age	1.02	(1.00–1.04)	0.012	1.04	(1.00–1.07)	0.03	1.07	(0.99–1.04)	0.07
Schooling	1.02	(0.99–1.04)	0.16	0.99	(0.94–1.04)	0.77	1.03	(0.99–1.06)	0.08
Length of stay in ED	1.00	(1.00–1.00)	0.006	1.00	(0.99–1.07)	0.31	1.00	(1.00–1.01)	0.012
Referral to hospitalization	3.12	(2.29–4.24)	< 0.001	–	–	–	–	–	–
Geriatric care trained residents model (Hospital A final)	0.66	(0.46–0.96)	0.031	0.79	(0.39–1.61)	0.53	0.65	(0.42, 1.01)	0.05
FRAIL (dichotomic) (≥2)	1.34	(1.02–1.76)	0.033	0.80	(0.46–1.38)	0.42	1.59	(1.15–2.18)	0.005

^a^Same model than Table 3 without grip strength or MMSe and including Frail

## Discussion

This study demonstrates high mortality among older persons (21.7%) in two Mexican public general, non-specialized, hospitals and it reveals that 71% of the total deaths occurred during hospitalization after a visit to, and referral from, the ED. An associated factor to mortality that appears to be important is the length of stay in the ED. In all analysis, this variable was statistically significant and it may be explained as the presence of inappropriate processes of care at the ED. When a simplified Cox model was executed, hand grip strength was significant only for the group referred to hospitalization. It is plausible to think that patients referred to hospitalization were deteriorated and functionality was seriously affected.

The residents trained in geriatric care intervention seemed to be a protective factor in the last model (Table 4) when all patients were analyzed, and it is also significant for those patients referred to hospitalization. In this report, the training model was incorporated as the only other associated factor. Therefore, and in spite of the evident methodological limitations to evaluate the impact of such an intervention, it is possible to conclude that geriatric training of emergency and internal medicine residents is of the most importance.

Utilization patterns of the ED and the hospitalization of older adults are different from those of other age groups, primarily because of multimorbidity, frailty, cognitive impairment, depression, and poor self-rated health [28, 29]. It has been argued that older adults are the main cause of the exponential increase in health care costs [30]. However, there are many reasons beyond demographic transition which do not only involve cultural factors and clinical variables, but also involve health staff resistance to change, ageism, inadequate or incomplete geriatric training, and poor accessibility to emergency services [31], among others. A remarkable effort has been made by different agencies and societies worldwide to increase the knowledge and geriatric competencies of health professionals. However, effective interventions to insert the geriatric perspective in health staff are not easy and there is a lack of evidence of interventions [32].

Several studies have examined the deleterious effects of length of stay in the emergency department and hospitals with respect to the functionality and quality of life of older adults [33]. In this context, our findings revealed that in addition to the general traditional factors associated with mortality (age and education level), geriatric conditions (delirium, cognitive impairment, multi-medication, and frailty) play an important role for older adults being at risk of death. Our findings are consistent with the results of previous reports [6, 10, 34]. A delay in seeking attention at the ED and the time spent in the ED were variables that correlated significantly with mortality; both maintain significance for the group of participants referred for hospitalization in the Cox regression adjusted for all significant variables (see Table 2). Length of stay at the ED was relevant to participants that were referred for hospitalization. Although it could be argued that this finding represents a selection bias, these variables reflect serious difficulties in providing emergency care. The variables also reflect a deferral of attention given to specific groups of patients and the inability of health staff to identify patients at risk in a timely and accurate manner [35]. As highlighted by Adams and Gerson [36], the current model of ED care was designed for acutely ill and injured patients, not slow-moving, functionally affected geriatric patients with multimorbidity.

We believe that our results are relevant and that the methodological components of the study were supervised carefully in spite of difficulties in the ED setting. We also believe that the aging research conducted in this country, Mexico, is important and could be useful to countries facing similar situations where chaos in the ED is usual. Of course, our study has some methodological limitations. Patients that were not admitted to the ED may represent a selection bias, underestimating the effect if those patients were seriously ill and transferred to another hospital.

Finally, frailty appears to be an important variable associated with mortality, as proven to be for other populations and a wide variety of settings. Proper attention given to frail persons in EDs is a pivotal issue. Detecting frailty status is crucial and the development of standard care protocols is an important area of research [37].

Creating public policies that support funding for developing quality patient care strategies and gathering scientific evidence, as well as promoting structural adjustments in health care institutions (to care for the needs of older adults), should be encouraged.

Many developing countries and emerging economies face major challenges in a bid to effectively meet the health care needs of a growing older population. Mexico is not an exception in this situation. Aging populations and fragmented health care and social systems combine to provide an inappropriate response to this new scenario.

## Conclusions

Mortality in patients over 60 years old admitted to the ED is high. Factors of mortality are related to the organization of health services and the patient’s length of stay in the ED. Also, hand grip strength as a key component of frailty seems to be an important associated factor. The health care system must quickly adjust its response to an aging population and restructure health services to better meet the needs of older individuals. This must be a priority.

## Abbreviations

CAM: Confusion Assessment Method
CI: Confidence interval
ED: Emergency department
HR: Hazard ratios
IMSS: Instituto Mexicano del Seguro Social
IQR: Interquartile range
ISAR: Identification of Seniors at Risk
KMC: Kaplan-Meier curves
MMSE: Mini-Mental State Examination
SAGE: Study on Global Ageing and Adult Health
SD: Standard deviation
